# Environmental and host determinants of acute gastric dilatation in nonhuman primates: a retrospective cohort study

**DOI:** 10.3389/fvets.2026.1936116

**Published:** 2026-08-27

**Authors:** Gyu-Seo Bae, Eunsu Jeon, Joonwon Mo, Ju-Yeon Lee, Dong-Ho Lee, Jung Joo Hong, Jinsoo Lee, Bon-Sang Koo

**Affiliations:** 1 National Primate Research Center, Korea Research Institute of Bioscience and Biotechnology, Cheongju, Republic of Korea; 2 Laboratory Animal Medicine, College of Veterinary Medicine, Chungnam National University, Daejeon, Republic of Korea; 3 Primate Resources Center, Korea Research Institute of Bioscience and Biotechnology, Jeongeup, Republic of Korea; 4 KRIBB School of Advanced Bioconvergence, Korea University of Science and Technology, Daejeon, Republic of Korea; 5 KRIBB School of Bioscience, Korea University of Science and Technology, Daejeon, Republic of Korea

**Keywords:** acute gastric dilatation, housing type, macaque, non-human primates, stress

## Abstract

Acute gastric dilatation (AGD) has been occasionally reported as a clinically important condition in laboratory non-human primates, with potentially fatal outcomes. However, the environmental and host-related factors contributing to AGD occurrence remain poorly understood. In this study, we investigated AGD cases identified at two independent primate centers in Republic of Korea and assessed their associations with environmental and host-related factors. In univariable analyses, housing type, facility location, and sex were significantly associated with AGD occurrence. In the exploratory multivariable Firth analysis, a statistically significant association was observed only for housing type; however, the independent contribution of each factor could not be clearly determined because of the small number of AGD cases and substantial confounding among these variables. AGD-affected animals may exhibit stress-associated leukocyte changes, characterized mainly by eosinopenia, mild lymphopenic tendencies, and monocytosis in a subset of animals. In the blood chemistry analysis, alanine aminotransferase showed an overall upward distribution and serum creatinine values tended to be lower. In conclusion, the findings suggest a potential association between individual housing and AGD occurrence, while stress-related physiological changes and reduced locomotor activity may represent plausible hypotheses underlying this association, although these mechanisms were not directly assessed in the present study.

## 1 Introduction

Acute gastric dilatation (AGD) is a sporadic but potentially fatal gastrointestinal emergency in laboratory-housed or captive non-human primates (NHPs) (1). Previous studies reported that AGD may account for approximately 3.5–12% of mortality depending on the population and denominator examined, with reported case fatality rates approaching 80% (2–4). Owing to its rapid progression, affected animals often die before overt clinical signs are recognized, and many cases are therefore diagnosed only at necropsy. Therefore, identifying potential risk factors and developing preventive strategies are critical for improving health management in captive NHP populations (1, 5, 6). AGD is not limited to Old World primates but has also been reported as a cause of sudden death in New World primates. These observations suggest that AGD should be regarded as an important health concern across a broad range of NHPs species (6).

AGD is caused by gastric distension resulting from the rapid accumulation of intragastric gas. The resulting gastric enlargement may compress the diaphragm and increase intra-abdominal pressure, thereby contributing to respiratory and circulatory dysfunction and, in severe cases, gastric rupture (7). Although direct evidence of these systemic consequences in NHPs is limited, AGD in humans has been reported to induce acute respiratory distress and circulatory compromise through diaphragmatic compression and elevated intra-abdominal pressure (8). Furthermore, in other animal species, particularly dogs with gastric dilatation-volvulus (GDV), acute gastric distension has been associated with cardiovascular alterations, including reduced venous return, shock, myocardial dysfunction, and cardiac arrhythmias (9). These findings suggest that AGD may involve shared pathophysiological mechanisms across species, contributing to the rapid worsening of clinical signs (10). However, findings from humans and canine GDV may not be directly applicable to AGD in NHPs because of differences in anatomy, pathological manifestations, and clinical context. Therefore, cross-species extrapolation should be made with caution.

Although the etiology of AGD remains incompletely understood, previous reports have suggested that gas-producing bacteria, including *Sarcina ventriculi*, have been implicated in gastric fermentation and intragastric gas accumulation. Such organisms have been reported in gas-associated gastrointestinal lesions, including gastrointestinal bloat in livestock and emphysematous gastritis in humans (11, 12). *Sarcina*-like organisms have previously been reported in NHPs with AGD (7, 13). However, these observations are insufficient to establish *Sarcina* as a direct causative agent of AGD. Because no microbiological investigation was performed in the present study, this information is presented solely as background based on previous reports and should be regarded as hypothesis-generating rather than evidence of a causal role in AGD.

Beyond microbiological factors, non-infectious factors, including housing and social conditions, may influence host-related factors such as stress responses, gastrointestinal motility, and immune status, thereby contributing to disease susceptibility (14). Previous studies in macaques have shown that relocation or social separation from group to individual housing can induce transient glucocorticoid responses and changes in locomotor activity, while paired or social housing may reduce stress-related behaviors compared with single housing (15–17). Stress-related physiological changes may affect gastrointestinal motility, secretion, barrier function, and immune responses, thereby altering the intestinal environment (18). Reduced locomotor activity may also slow gastrointestinal motility and change gut transit time, which can influence microbial composition and metabolism (19, 20). However, there is very limited evidence from systematic studies examining the relationship between environmental conditions and AGD occurrence in NHPs.

In this study, we conducted a retrospective cohort analysis to identify environmental and host-related factors associated with AGD in captive cynomolgus and rhesus macaques. We hypothesized that housing environment, host characteristics, and associated stress-related factors may influence susceptibility to AGD. By assessing the associations among these factors and AGD occurrence, this study aimed to improve our understanding of potential risk factors for AGD in these animal species. These findings may provide a basis for developing husbandry management strategies for AGD prevention and veterinary interventions, thereby contributing to more systematic management and treatment strategies for NHPs.

## 2 Materials and methods

### 2.1 Animals

In this study, we retrospectively analyzed health monitoring records from cynomolgus macaques and rhesus macaques housed at the National Primate Research Center (NPRC) in Ochang and the Primate Resources Center (PRC) in Jeongeup, Republic of Korea. Both facilities are affiliated with the Korea Research Institute of Bioscience and Biotechnology (KRIBB). The dataset primarily included records obtained between 2023 and 2025 from the NPRC and records obtained in 2025 from the PRC. A total of 913 unique animals, comprising 724 cynomolgus monkeys and 189 rhesus monkeys, were included, with 1,227 health monitoring records available overall. These consisted of records from 232 cynomolgus monkeys and 38 rhesus monkeys housed at the NPRC in Ochang between 2023 and 2025, and records from 492 cynomolgus monkeys and 151 rhesus monkeys housed at PRC in Jeongeup in 2025. At the NPRC, repeated annual health assessments were available for some animals: 32 animals were assessed in both 2023 and 2024, 16 in both 2024 and 2025, and 133 in all 3 years, whereas only 2025 data were available from the PRC. To avoid treating repeated measurements as independent observations, the most recent health examination record for each animal was used for statistical analyses.

### 2.2 Housing conditions

All animals were maintained under specific pathogen-free (SPF) conditions, although housing environments differed between facilities. Cynomolgus and rhesus macaques at the NPRC in Ochang were housed in a strictly controlled indoor environment, with a temperature of 24 ± 2 °C, relative humidity of 50 ± 5%, light intensity of 150–300 lx, 10–20 air changes per hour, and a 12-h light/dark cycle. In contrast, animals at the PRC in Jeongeup were maintained in SPF-managed semi-open housing facilities under routine husbandry conditions. A commercial primate diet (2050 Teklad Global 20% Protein Primate Diet; Harlan, Envigo, UK) was provided as the basal diet, and water was available ad libitum. Detailed information on environmental enrichment, handling frequency, stocking density, and other husbandry practices was limited; therefore, potential differences in these factors between facilities cannot be excluded as additional confounding factors.

Group housing was maintained as the standard housing condition, and animals were kept in enclosures that met the recommended minimum space requirements for NHPs housed in pairs or groups, according to the *Guide for the Care and Use of Laboratory Animals*. Individual housing was used only when scientifically or veterinary justified, including during pre-experimental acclimation and experimental procedures, for animals requiring protection from conspecific aggression, for highly aggressive animals, for veterinary treatment, or for quarantine before transfer. The dimensions of the group housing facilities varied according to group size; for example, enclosures measured 3,150 × 1,450 × 2,700 mm (W × L × H) for four animals and 4,600 × 2,450 × 2,700 mm (W × L × H) for eleven animals. When singly housed, animals were kept in cages measuring 760 × 750 × 860 mm (W × L × H), which met the Group 4 (<15 kg) standards specified in the *Guide for the Care and Use of Laboratory Animals* (14). Environmental enrichment included the provision of seasonal fruits as food-based enrichment, together with various manipulable enrichment devices and toys. All experimental procedures were performed after review and approval by the Institutional Animal Care and Use Committee (IACUC) of the Korea Research Institute of Bioscience and Biotechnology (approval numbers: KRIBB-AEC-23063, KRIBB-AEC-24019, and KRIBB-AEC-25022), in compliance with all applicable regulations and guidelines.

### 2.3 AGD diagnosis and risk factor analysis

AGD was defined using uniform diagnostic criteria across both institutions throughout the study period. AGD was diagnosed based on a comprehensive assessment of clinical signs, including acute abdominal distension, anorexia, decreased activity, and shock, together with radiographic evidence of marked gastric distension containing gas, fluid, and/or ingesta (1, 21). In animals that died or were found dead, the diagnosis was further confirmed by necropsy findings, including markedly enlarged stomach filled with gas, water, and ingesta with or without gastric rupture (13, 22, 23). During the study period, a total of 10 AGD cases were identified in eight animals, of which seven cases were fatal or identified after the animals were found dead. Environmental and host-related factors were extracted from health monitoring records and analyzed for their associations with AGD occurrence. These variables included facility location, building, housing type, animal species, age, body weight, and sex. Housing type was recorded based on the animal’s housing status at the time of AGD onset, and all animals had been maintained under the same housing type for at least 2 weeks before onset.

### 2.4 Hematology and blood chemistry

After an overnight fast, blood samples were obtained from the femoral vein under ketamine hydrochloride anesthesia (10 mg/kg). Blood was collected into EDTA-K₂ tubes for hematological analysis and sodium heparin tubes for blood chemistry analysis. Plasma was separated by centrifugation at 1,600 × g for 15 min. Hematological parameters were measured using a HEMAVET 950FS analyzer (Drew Scientific Ltd., USA) in NPRC and a ADVIA 2120i analyzer (Siemens, Germany) in PRC, whereas plasma biochemical parameters were analyzed using a Dri-Chem 7000i biochemistry analyzer (Fujifilm, Japan) in NPRC and PRC. The results were interpreted using species- and sex-specific reference values previously established by our institution (24). The interval between blood collection and AGD onset ranged from 3 to 330 days; in all but one case, blood samples were collected more than 40 days before AGD onset (Table 1).

**Table 1:** Summary of acute gastric dilatation (AGD) cases in non-human primates.

Animal species	Sex	Body weight (kg)	Age at onset (years)	Date of AGD occurrence	Clinical outcome	Date of death	Date of blood collection	Blood collection–AGD interval (days)	Location
Rhesus	M	6.59	2008.8.14	2023.05.10	Death	2023.05.10	2022.06.14	330	Ochang
Cyno	F	3.25	2017.04.01	2023.06.13	Death	2023.06.13	2022.08.31	286	Ochang
Cyno	M	4.15	2018.9.19	2024.03.13	Death	2024.03.13	2023.06.27	260	Ochang
Rhesus	M	5.02	2015.11.07	2024.03.26	Survival	–	–	–	Ochang
				2024.05.14	Survival	–	–	–	
				2024.06.16	Death	2024.06.16	2024.06.13	3	
Cyno	M	4.12	2016.05.04	2024.07.28	Death	2024.07.30	2024.06.11	47	Ochang
Cyno	M	2.88	2019.09.21	2025.08.11	Death	2025.08.11	2025.07.01	41	Ochang
Cyno	F	2.85	2019.12.04	2025.11.12	Death	2025.11.12	2025.07.07	128	Ochang
Cyno	M	3.65	2015.1.15	2025.11.10	Survival	-	2025.08.19	83	Jeongeup

Cyno: cynomolgus macaque, M: Male, F: Female.

### 2.5 Statistical analysis

A retrospective cohort analysis was conducted to assess factors associated with AGD occurrence during the observation period. Animals that did not develop AGD during the study period were considered the control population for epidemiological analyses. For hematological and biochemical comparisons, animals with concurrent gastrointestinal disorders or other clinically apparent systemic diseases that could affect laboratory parameters were excluded from the control group.

Given the rarity of AGD and the small number of events in some comparison groups, analyses included descriptive comparisons and Firth penalized logistic regression to account for rare events and potential separation. AGD occurrence proportions were calculated for each category of housing type, facility location, sex, and animal species, and group differences were assessed using Fisher’s exact test. Wilson 95% confidence intervals were calculated for occurrence proportions. Univariable Firth penalized logistic regression was performed to estimate odds ratios (ORs) and 95% confidence intervals for the factors evaluated. An exploratory multivariable Firth penalized logistic regression model including housing type, facility location, and sex was also performed to evaluate their associations with AGD when considered simultaneously. Given the limited number of AGD events, the multivariable analysis was considered exploratory and interpreted with caution. Age and body weight were compared between AGD and non-AGD animals using the Mann–Whitney U test. Potential structural associations among major covariates, including facility location, housing type, and sex, were evaluated using cross-tabulation, within-row proportions, and Fisher’s exact test with Monte Carlo simulation where appropriate. For hematological and blood chemistry analyses in AGD-affected animals, z-scores were calculated using species- and sex-specific reference means and standard deviations. Values with |z| > 2 were interpreted as being outside the expected reference range. Stress-associated leukocyte changes were further assessed using z-scores for eosinophils, monocytes, neutrophils, and lymphocytes. Statistical analyses were performed in R, and *p*-values <0.05 were considered statistically significant.

## 3 Results

### 3.1 AGD occurrence according to host and environmental factors

**Figure 1:**
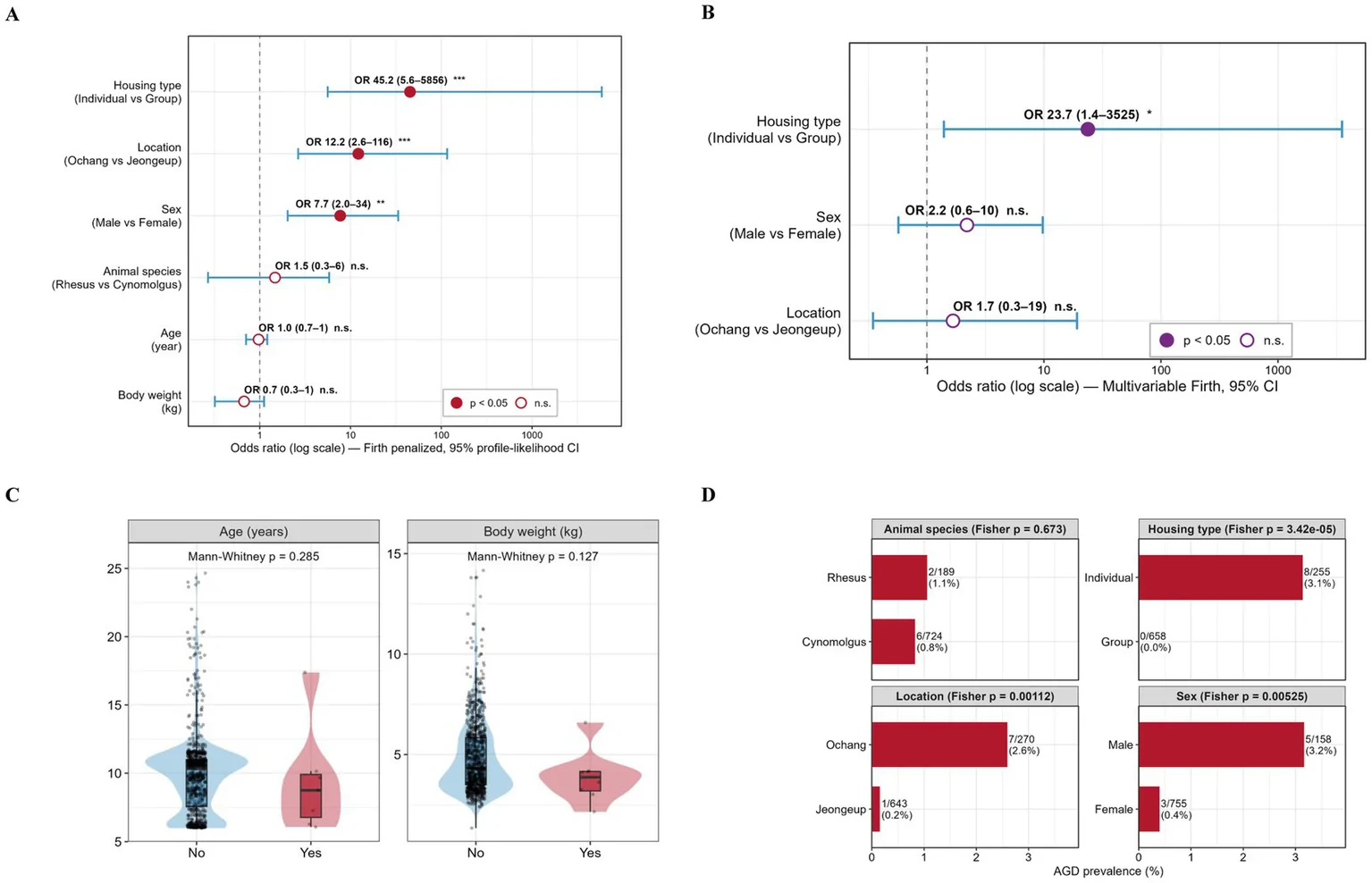
Environmental and host-related factors associated with AGD occurrence in non-human primates. **(A)** Univariable Firth penalized logistic regression was performed to estimate odds ratios (*ORs*) and 95% confidence intervals for housing type, facility location, sex, animal species, age, and body weight. **(B)** An exploratory multivariable Firth penalized logistic regression model including housing type, facility location, and sex was used to estimate adjusted *ORs* and 95% confidence intervals. Given the limited number of AGD events, the multivariable estimates should be interpreted with caution. **(C)** Age and body weight distributions between AGD and non-AGD animals were compared using the Mann–Whitney *U* test. **(D)** AGD prevalence was compared according to animal species, housing type, facility location, and sex using Fisher’s exact test.

**Figure 2:**
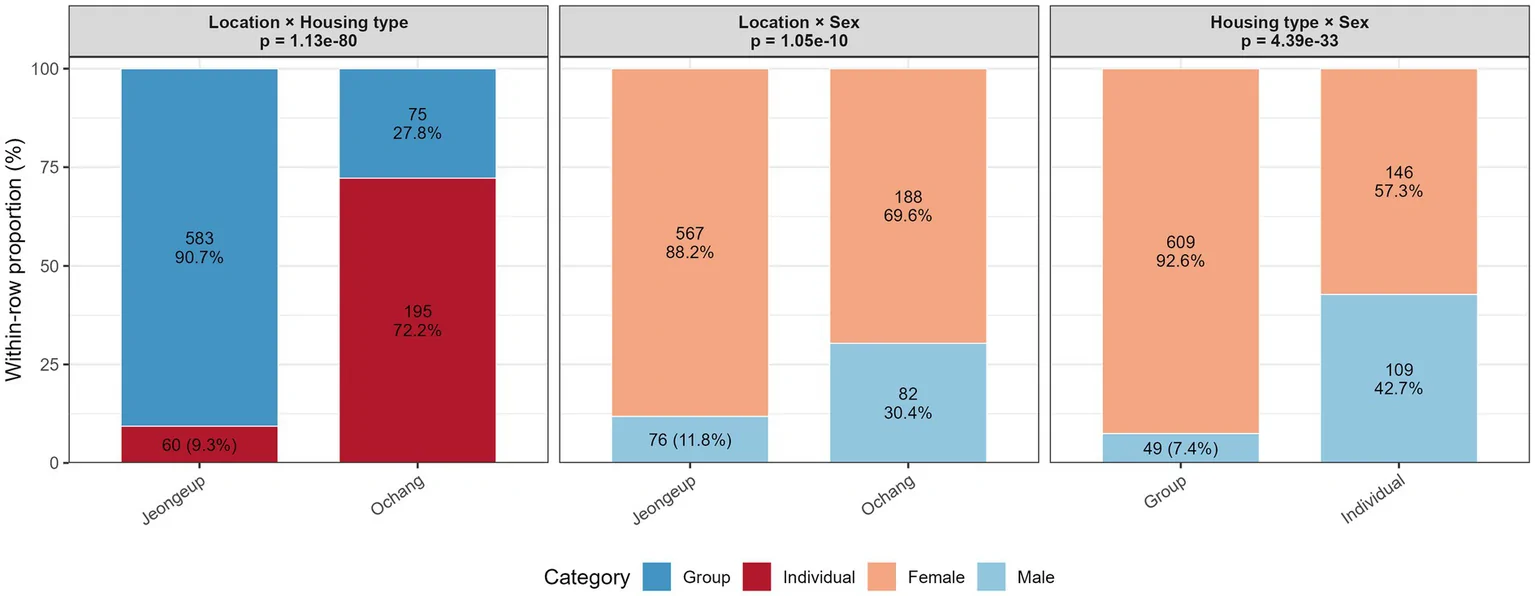
Structural associations among significant environmental and host-related factors associated with AGD occurrence. Stacked bar plots show the within-row proportions for each pairwise comparison: facility location by housing type, facility location by sex, and housing type by sex. Labels indicate the number and percentage of animals in each category. Fisher’s exact test was used to evaluate pairwise associations among variables, showing that these factors were not independently distributed across the study population.

During the study period, a total of ten AGD cases were identified. Among the 10 AGD episodes identified during the study period, three recurrent episodes occurred in one animal (Table 1). For subsequent analyses, these recurrent episodes were counted as a single case. Univariable Firth penalized logistic regression showed significant associations of AGD occurrence with housing type, facility location, and sex, whereas animal species, age, and body weight were not significantly associated with AGD (Figure 1A). In the exploratory multivariable Firth analysis including housing type, facility location, and sex, only housing type remained statistically significant (Figure 1B). Consistent with the Firth regression results, nonparametric and descriptive comparisons showed no significant differences in age or body weight between AGD and non-AGD animals based on the Mann–Whitney U test (Figure 1C). Similarly, comparison of AGD occurrence proportions using Fisher’s exact test showed differences according to housing type, facility location, and sex, but not animal species (Figure 1D). The occurrence rates were 1.1% in rhesus macaques and 0.8% in cynomolgus macaques (Figure 1D). However, because AGD was a rare event and the number of cases was small, several estimated associations had wide confidence intervals, indicating limited precision; therefore, these results should be interpreted with caution (Figures 1A,B). Structural association analysis showed that facility location, housing type, and sex were not evenly distributed across the study population (Figure 2). These findings indicate that the associations of AGD occurrence with location, housing type, and sex may be mutually confounded and that the associations observed in the univariable analyses should therefore not be interpreted as independent effects (Figure 2).

### 3.2 AGD occurrence according to hematological values

**Figure 3:**
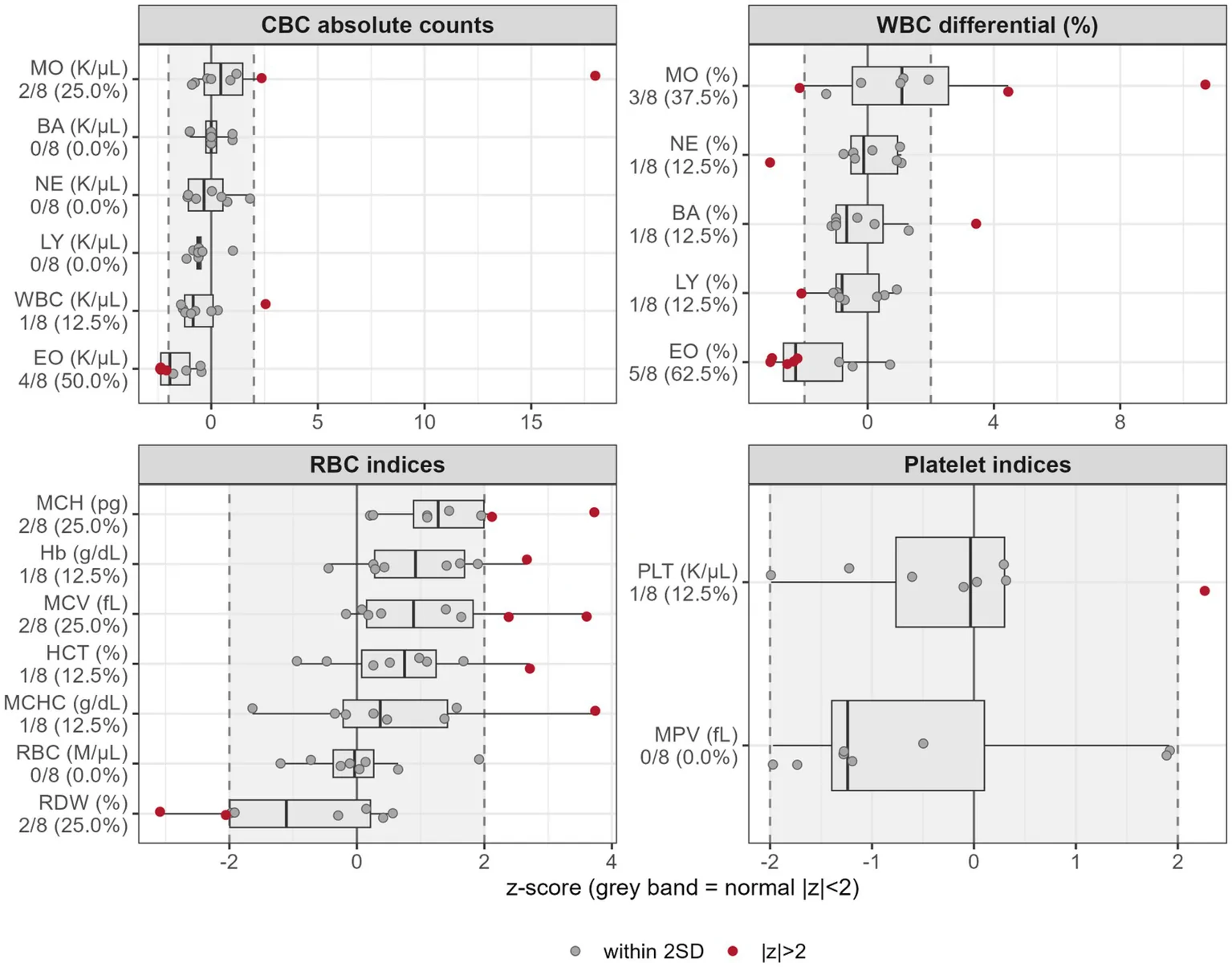
Hematological profiles in AGD-affected non-human primates based on *z*-score analysis. Hematological parameters were converted to *z*-scores using species- and sex-specific reference values and grouped into CBC absolute counts, WBC differential percentages, RBC indices, and platelet indices. The gray shaded area indicates values within ±2 standard deviations (SDs) of the reference mean. Gray points indicate values within ±2 SDs, whereas red points indicate values outside this range (|*z*| > 2). Box plots summarize the distribution of *z*-scores for each parameter. Values shown below each parameter indicate the number of animals with |*z*| > 2 relative to the number of animals with available measurements, with the corresponding percentage [*n*/*N* (%)].

**Figure 4:**
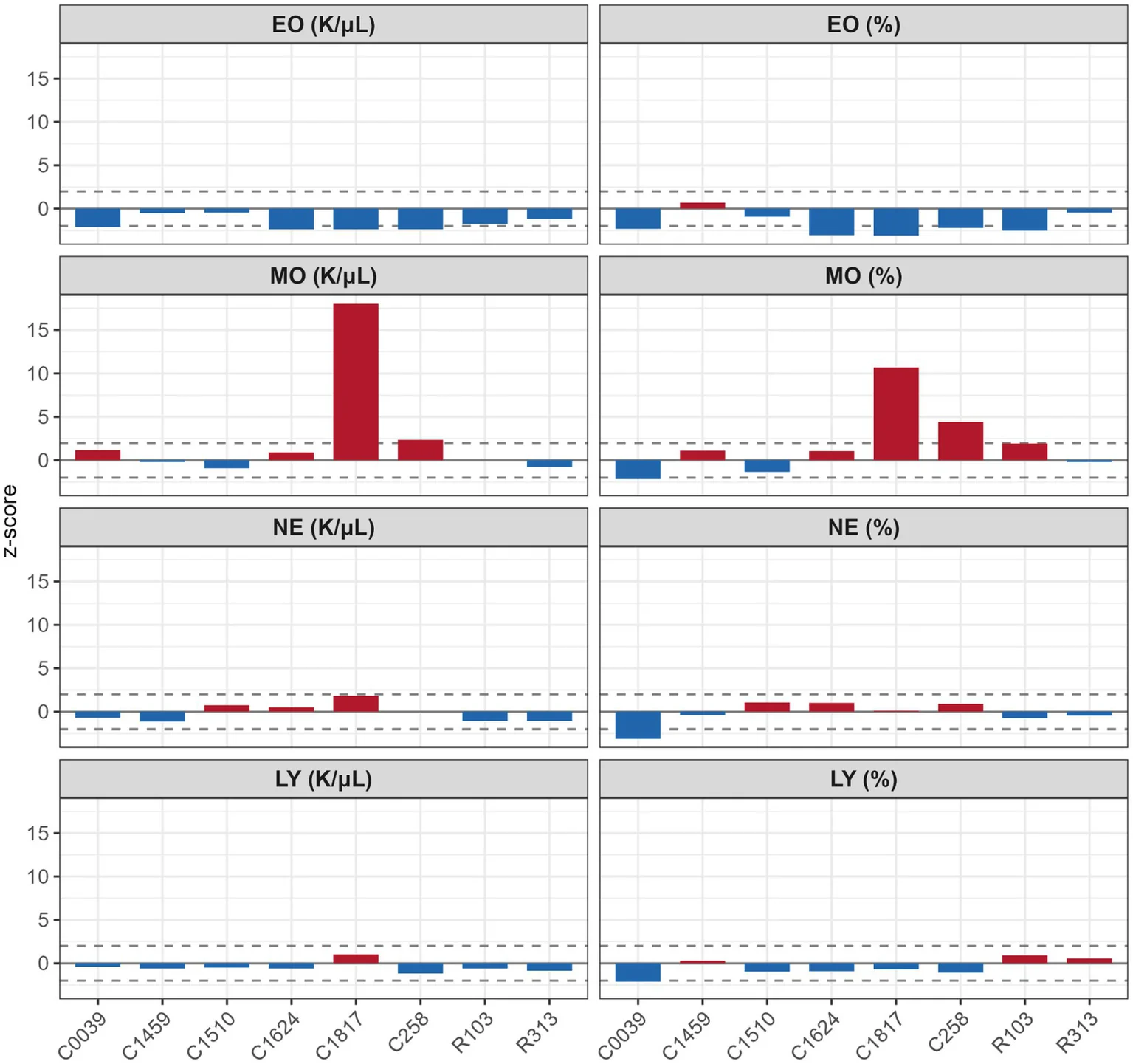
Individual-level leukocyte differential changes in AGD-affected non-human primates based on z-score analysis. Absolute counts and relative percentages of eosinophils (EO), monocytes (MO), neutrophils (NE), and lymphocytes (LY) were converted to z-scores using species- and sex-specific reference values. Each bar represents the z-score of an individual animal. Dashed horizontal lines indicate ±2 standard deviations from the reference mean. Red bars indicate positive z-scores, and blue bars indicate negative z-scores.

Hematological parameters were further assessed using z-scores calculated from species- and sex-specific institutional reference values derived from cynomolgus and rhesus monkeys aged 48–96 months maintained at the Ochang facility (24). Despite the relatively long interval between blood collection and AGD onset, which was at least 41 days in all but one animal, eosinophil parameters showed an overall downward shift relative to the reference values (Table 1; Figure 3). Several animals had eosinophil z-scores below −2, indicating values outside the expected reference range. In addition, lymphocyte and total WBC counts showed a mild downward tendency, although most values remained within the |z| ≤ 2 range. The numbers and proportions of animals with |z| > 2 for each parameter are presented in Figure 3. Stress leukogram analysis showed a consistent downward tendency in eosinophil z-scores among AGD-affected animals (Figure 4). Lymphocyte z-scores also tended to be lower overall, although most values remained within the expected reference range. In contrast, monocyte z-scores were increased in some animals, including one animal with a marked elevation. These findings indicate leukocyte changes compatible with previously described stress-associated patterns, characterized mainly by eosinopenia, mild lymphopenic tendencies, and monocytosis in a subset of animals (Figure 4).

### 3.3 AGD occurrence according to blood chemistry values

**Figure 5:**
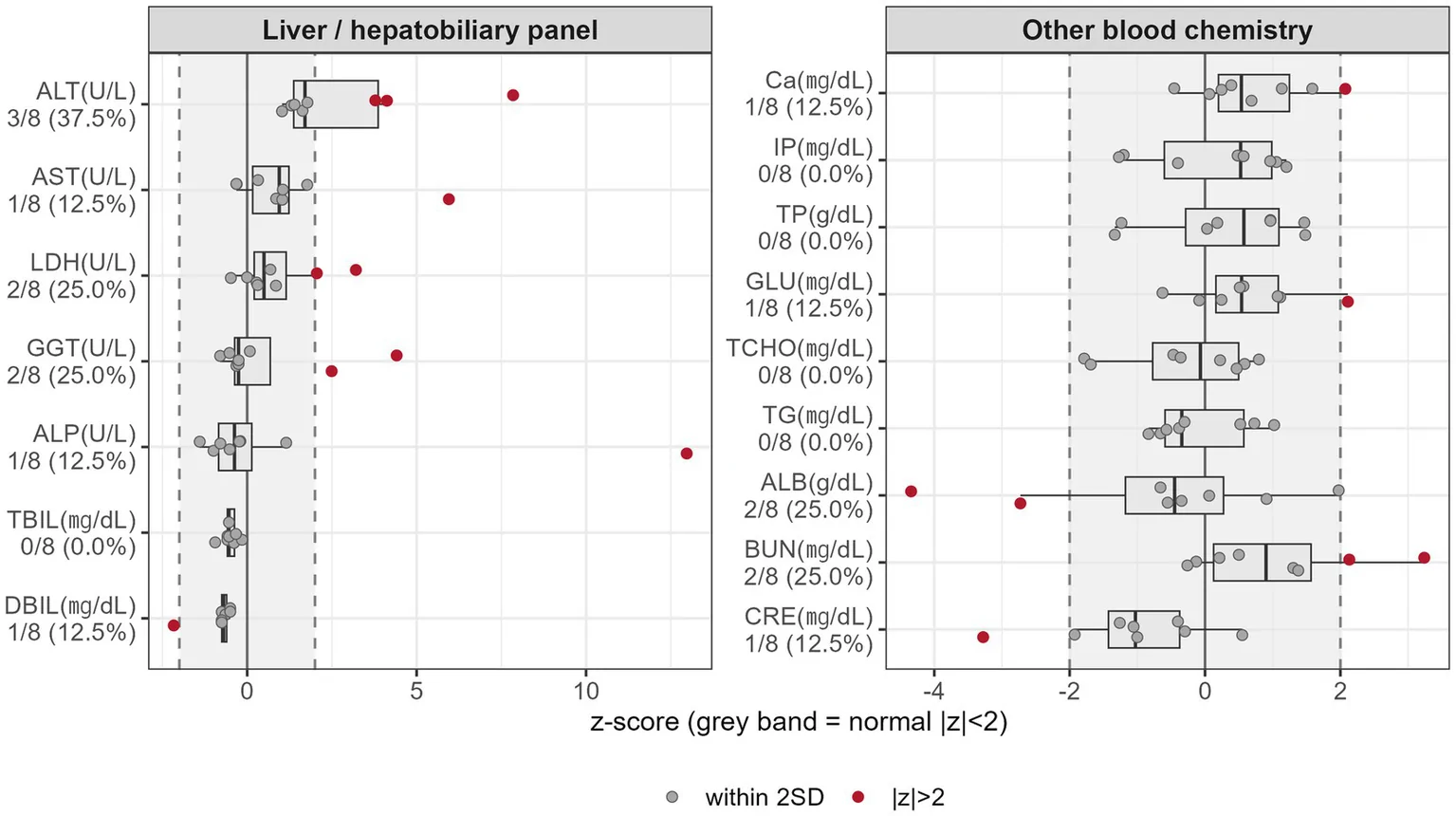
Blood chemistry profiles in AGD-affected non-human primates based on *z*-score analysis. Blood chemistry values were standardized using species- and sex-specific reference values and categorized into liver/hepatobiliary and other blood chemistry panels. The gray shaded area represents the reference range within ±2 *SD*s. Red points indicate values exceeding this range (|*z*| > 2), highlighting deviations from the corresponding reference population. Values shown below each parameter indicate the number of animals with |*z*| > 2 relative to the number of animals with available measurements, with the corresponding percentage [*n*/*N* < (%)].

In the blood chemistry analysis, z-scores were likewise calculated using the species- and sex-specific institutional reference values. Alanine aminotransferase (ALT) showed an overall upward distribution relative to the species-specific reference values, while aspartate aminotransferase (AST) and lactate dehydrogenase (LDH) also tended to be higher in some AGD-affected animals, with several values exceeding the |z| > 2 threshold (Figure 5). Among the other biochemical parameters, blood urea nitrogen (BUN) values were generally distributed above the reference mean, whereas creatinine values tended to be lower. This pattern suggests a relative increase in BUN compared with creatinine. The numbers and proportions of animals with |z| > 2 for each biochemical parameter are presented in Figure 5.

## 4 Discussion

AGD is a clinically important condition in laboratory NHPs, with potentially fatal outcomes. Historical colony studies have reported that AGD accounted for approximately 3.5–12% of mortality, whereas AGD was identified in 8 of 913 animals (0.9%) in the present study, with occurrence rates of 1.1% in rhesus macaques and 0.8% in cynomolgus macaques (2–4). Although these estimates are not directly comparable because of differences in study populations and denominators, they provide epidemiological context for the occurrence of AGD in NHP colonies. However, the environmental and host-related factors contributing to AGD occurrence remain poorly understood. In this study, we investigated AGD cases identified at two independent primate centers and assessed their associations with environmental and host-related factors.

In the present study, housing type, facility location, and sex were significantly associated with AGD occurrence in the univariable analyses. In contrast, age, body weight, and animal species were not significantly associated with AGD (Figure 1). However, structural association analysis showed that housing type, facility location, and sex were not independently distributed across the study population (Figure 2). Individual housing was more common at the Ochang facility than at the Jeongeup facility, and males were more frequently represented among individually housed animals. Together with the wide confidence intervals resulting from the small number of AGD cases, these findings suggest that the observed associations should be interpreted cautiously and that the independent contribution of each factor could not be clearly determined. Nevertheless, within the limitations of this study, housing type, facility location, and sex were identified as factors that differed significantly according to AGD occurrence. In the exploratory multivariable Firth analysis including these three factors, a statistically significant association was observed only for housing type; however, this finding should be interpreted cautiously given the small number of AGD cases and substantial confounding among the variables.

Nevertheless, biologically possible explanations can be proposed for the observed associations of AGD occurrence with housing type, facility location, and sex. The difference observed between group and individual housing may partly reflect differences in the available activity space and opportunities for spontaneous physical activity (14). Group-housing environments may provide NHPs with a broader behavioral range and greater opportunities for locomotion than individual cages, and social housing is recommended as the default housing condition for social NHPs to support species-typical behavior (14). Reduced locomotor activity may impair gastrointestinal motility and alter gut transit time, thereby influencing microbial composition and metabolism (20). Similarly, in human patients, *Sarcina ventriculi* has been reported in association with delayed gastric emptying and gastric outlet obstruction, suggesting that gastric stasis may favor the colonization or proliferation of gas-producing organisms (25, 26). In dogs, reduced gastrointestinal motility has been reported to contribute to gastric dilatation-volvulus by impairing gastric motor function and delaying gastric emptying (27). Therefore, reduced opportunities for locomotor activity in individual housing may be hypothesized as a potential factor associated with AGD occurrence.

Previous studies in macaques have shown that individual housing may increase stress-related behaviors and glucocorticoid responses, while pair or social housing can reduce stereotypic behaviors and provide social buffering (16, 28, 29). Physiological assessments of stress in macaques have included HPA-axis markers such as plasma or serum cortisol, adrenocorticotropic hormone, fecal glucocorticoid metabolites, and hair cortisol, although the effects of housing conditions on these markers have not been consistent across studies (30, 31). Stress-related activation of corticotropin-releasing factor (CRF) signaling and autonomic pathways has been shown to inhibit upper gastrointestinal motor function and delay gastric emptying (32, 33). Therefore, stress associated with individual housing may impair gastric motor function and promote delayed gastric emptying or gastric stasis, which could provide favorable conditions for gas-producing organisms such as *Sarcina ventriculi*. Although this mechanism was not directly examined in the present study, it may provide a biologically plausible explanation for the higher AGD occurrence observed in individually housed animals.

Furthermore, sex-related differences in activity budgets have been reported in macaques, with males showing greater moving or social activity than females in some populations (34). Male macaques are generally larger and heavier than females, and larger animals require greater space allowance under single-cage housing conditions. Therefore, males may have experienced relatively greater spatial restriction than females under the same individual housing conditions (14, 35). Therefore, under the same individual cage conditions, males may have been more affected by spatial and behavioral restriction than females, leading to greater limitations in locomotor activity and possibly stronger stress-related physiological responses. This may represent one possible explanation for the observed association between male sex and AGD occurrence.

The observed eosinopenia, mild lymphopenic tendency, and monocytosis are compatible with patterns previously described in stress- or corticosteroid-associated leukograms, which commonly include lymphopenia, eosinopenia, and, in some species, monocytosis (Figure 4) (36, 37). In addition, ALT tended to increase, whereas AST and LDH showed little elevation, suggesting possible mild hepatocellular change rather than generalized tissue injury (38). Because serum creatinine production is largely related to muscle mass, the tendency toward lower creatinine may suggest differences in muscle mass or lean body condition (39). In individually housed animals, this finding could be partly related to reduced locomotor activity and chronic stress-associated catabolic changes; species- and sex-however, these interpretations remain speculative because stress markers, muscle condition, and physical activity were not directly assessed in the present study. Notably, except for one animal, blood samples were collected more than 1 month before AGD onset. Given the relatively long interval between blood collection and AGD onset, it remains unclear whether these findings represent predisposing physiological characteristics associated with AGD or unrelated individual variation. Additionally, limited information on the environmental and housing conditions of the reference population may have affected the comparability of the institutional reference values with the study population. Therefore, they should not be interpreted as immediate preclinical changes or direct evidence of AGD pathogenesis.

Overall, this study evaluated host- and environment-related factors associated with AGD occurrence in NHPs. The observed association between individual housing and AGD may raise the hypothesis that stress-related physiological changes and reduced locomotor activity could influence gastrointestinal motility and thereby contribute to AGD susceptibility. However, several important limitations should be considered. The proposed mechanisms remain speculative, as behavioral activity, stress biomarkers, gastrointestinal motility, and microbiological changes were not directly assessed. In addition, individual housing may be associated with other management or clinical factors, such as quarantine, postoperative recovery, experimental procedures, behavioral management, or veterinary treatment, which could act as potential confounding factors and were not systematically evaluated in this retrospective study. Detailed housing and environmental information for the reference population was limited, which may affect comparability with the study animals. The retrospective design may also have introduced potential information bias, and the small number of AGD cases limited the estimation of definitive risk factors. Therefore, the current findings should be interpreted as epidemiological associations and do not provide direct evidence for causal or mechanistic relationships between individual housing and AGD.

Despite these limitations, the observed associations with housing conditions and host characteristics may provide a basis for further investigation of factors potentially associated with AGD occurrence. The observed hematological and biochemical changes may also represent potential candidate biomarkers, although their predictive value requires prospective validation. Based on the factors suggested by the present findings, potential preventive strategies for individually housed NHPs may include enhanced environmental and behavioral enrichment, closer clinical and laboratory monitoring, and microbiological evaluation and appropriate management of potential gas-producing bacteria, such as *Sarcina ventriculi*. Further multicenter prospective studies incorporating behavioral monitoring, physiological stress biomarkers, gastrointestinal motility assessment, and microbiological characterization of the gastric environment are warranted to validate these associations, evaluate the predictive value of potential biomarkers, and clarify the mechanisms underlying AGD development.

## Data Availability

The raw data supporting the conclusions of this article will be made available by the authors, without undue reservation.
